# Patient-specific modeling of the volume of tissue activated (VTA) is associated with clinical outcome of DBS in patients with an obsessive-compulsive disorder

**DOI:** 10.1109/EMBC46164.2021.9630273

**Published:** 2021-11

**Authors:** Fuchang Jiang, Behzad Elahi, Mohit Saxena, Ilknur Telkes, Marisa DiMarzio, Julie G. Pilitsis, Laleh Golestanirad

**Affiliations:** Department of Biomedical Engineering, Northwestern University, Evanston, IL 60608 USA.; Department of Physical Therapy and Human Movement Sciences, Northwestern University, Chicago, IL 60611 USA.; Department of Radiology, Northwestern University, Chicago, IL 60611 USA.; Department of Neurosciences & Experimental Therapeutics, Albany Medical College, Albany, NY 12208 USA.; Department of Neurosciences & Experimental Therapeutics, Albany Medical College, Albany, NY 12208 USA.; Department of Neurosciences & Experimental Therapeutics, Albany Medical College, Albany, NY 12208 USA.; Department of Radiology and Department of Biomedical Engineering, Northwestern University, Chicago, IL 60611 USA.

## Abstract

Deep brain stimulation (DBS) promises to treat an increasing number of neurological and psychiatric disorders. DBS outcome is directly a factor of optimal targeting of the relevant brain structures. Computational models can help to interpret a patient’s outcome by predicting the volume of tissue activated (VTA) around DBS electrode contacts. Here we report results of a preliminary study of DBS in two patients with obsessive-compulsive disorder and show that VTA predictions, which are based on patient-specific volume conductor models, correlate with clinical outcome. Our results suggest that patient specific VTA calculation can help inform device programing to maximize therapeutic effects and minimize side effects.

## I. INTRODUCTION

Obsessive-compulsive disorder (OCD) is a debilitating disease with a lifetime prevalence of 2.3% [1]. Deep brain stimulation (DBS) —a neurosurgical procedure that involves implantation of electrodes in specific brain targets and sending electric pulses via an implanted pulse generator to these targets—is approved by the FDA to alleviate symptoms of refractory OCD. A variety of targets have been proposed, including anterior limb of the internal capsule (ALIC) [2], nucleus accumbens (NAcc) [3], and medial forebrain (MFB) [4]. Among these, stimulation of NAcc is shown to provide the largest improvement in patients’ Y-BOCS (Yale-Brown obsessive-compulsive scale) scores [5]. DBS is generally promising; however, patients’ outcomes have not always been consistent. One commonly accepted explanation for the variability in patient outcomes is the differences in stimulation that each patient receives. In this regard, computational models have been proposed to estimate the effect of stimulation on an individual basis [6–13]. In this study, we investigated if the volume of tissue activated (VTA) around the DBS electrode is associated with the patient’s clinical response to stimulation.

## II. METHODS

### A. Patients

Two patients (one male 54 y/o, one female 22 y/o) who were diagnosed with OCD underwent bilateral implantation of 8-contact DBS electrodes (Infinity-6172, Abbott Laboratories, Illinois, USA) in the ventral capsule/ventral striatum (VC/VS) region using a stereotactic Leksell frame and under local anesthesia. The following therapeutic stimulation parameters were used: monopolar configuration with contact 1 (left lead) and 9 (right lead) as the cathode and the implantable pulse generator (IPG) case as the anode, pulse width of 60 *μ*s, and pulse frequency of 160 Hz. The stimulation amplitude was 5.5 mA for patient 1 and 5.25 mA for patient 2. The impedance between the active electrode and the IPG was measured to be 926 Ω for both left and right electrode contacts in patient 1, and 1262 Ω and 1150 Ω for left and right electrodes, respectively, in patient 2.

Both patients underwent MRI in a 1.5 T Philips Ingenia scanner prior to their surgery, and whole-brain T_1_-weighted images were acquired with 1 × 1 × 1 mm^3^ isotropic resolution, TE= 4.5 ms, TR= 25 ms, and flip angle 30°. Postoperative CT images with thin cuts were acquired for electrode localization.

### B. Patient-specific models

The *headreco* module of SimNIBS [14] was used to segment T_1_-weighted MR images to create masks of scalp, skull, white matter, gray matter, CSF, and ventricles. Binarized 3D Volumetric segmentation of NAcc images was obtained using Freesurfer image analysis suite [15] and FSL [16].

Masks were smoothed in 3D slicer software [17] to remove sharp corners and extrusions smaller than 3 mm. The surface models were created for each compartment based on the smoothed masks. To reduce FEM simulation time, the total number of triangulated mesh elements were reduced by approximately 90% in Rhino 3D (Rhino, Robert McNeel & Associates for Windows, Washington DC, USA) using the build-in function *ReduceMesh*. All segmentation and model creation steps were performed in the patient’s native space.

**Figure 1.**
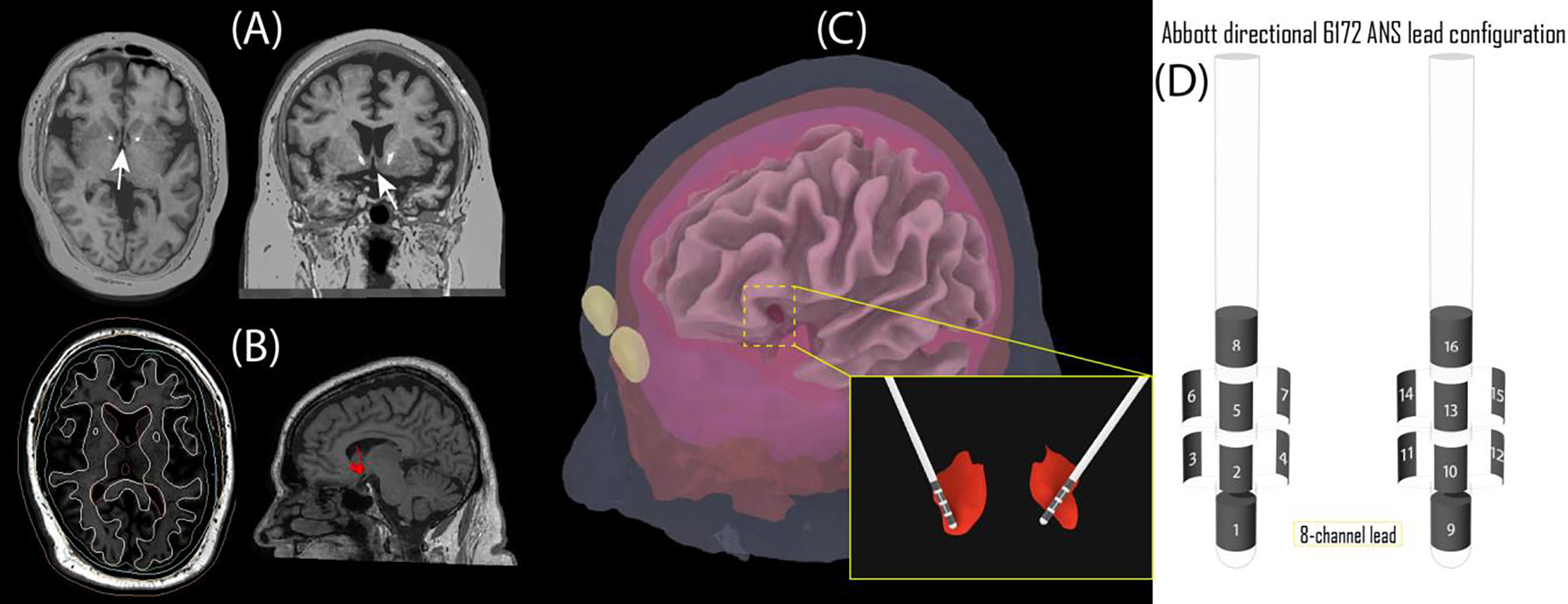
Steps of image segmentation and model creation. (A) post-op CT images were fused with pre-op MRI for electrode localization. (B) MRI images were segmented in SimNIBS to create masks of gray matter, white matter, CSF, skull, scalp, and ventricles. NAcc was segmented using Freesurfer and FSL. (C) 3D surfaces of different head compartments were created from smoothed masks. DBS leads were created around the centerline of lead artifact extracted from CT images. (D) Abbott directional 6172 ANS lead configuration.

To reconstruct the leads, postoperative CT images were first registered to the T_1_-weighted MRI (rigid registration, 6 degrees of freedom) using 3D Slicer’s BRAINS module [18]. The hyperdense electrode artifact was masked using thresholding tools, and a triangulated surface of the artifact was created and exported to Rhino 3D where the built-in function *LineThroughPt* was used to extract the centerline of the artifact. Finally, 3D surfaces of head compartments along with lines representing electrode trajectories were exported to ANSYS Maxwell 3D (ANSYS Inc. Canonsburg, PA) and models of Abbott directional lead 6172 ANS electrode array were created around lead line trajectories. The array consisted of two cylindrical electrodes and six segmented contacts. The position of the distal end of line trajectory was used as the starting point for the first electrode contact. Each contact had a height of 1.5 mm, a radius of 0.635 mm, and edge-to-edge inter-electrode spacing of 1.5 mm. Figure 1 shows steps of image segmentation and model creation.

### C. Finite element method (FEM) simulations

Maxwell 3D’s DC Conduction Solver was used to calculate the electric field potential *φ* throughout the model. The electric conductivities of different head components are given in Table I. We adjusted the conductivity of gray matter in each patient such that the electrode-IPG impedance in the simulation matched the clinical measurement. Current excitations were applied on the active electrode of each patient with the amplitude matching the clinical setting (5.5 mA for patient 1 and 5.25 mA for patient 2) with the bottom surface of the neck assigned as current sink to represent a distant return anode. The non-active contacts were assigned to 4e6 S/m conductivity as parts of the conductor model. The electrode shaft was considered as insulator with 0 S/m conductivity. Natural boundary condition was imposed between internal surfaces, which assured the normal component of the electric field displacement **D** had a jump when passing between surfaces of different objects which was equal with the superficial charge density. Neuman boundary condition was imposed on the outer surface of head.

To enhance the accuracy of field potentials close to electrode contacts, a cubic area of 20 mm × 20 mm × 20 mm was defined surrounding electrode contacts with enhanced mesh resolution.

ANSYS Maxwell was set to follow an adaptive mesh scheme where the mesh resolution was refined between iterative solutions until the calculated energy error fell below 0.08%. The final mesh had ~5 million tetrahedral elements, with the rms edge length of 0.4 mm inside the high-resolution mesh region and 0.1 mm on the active electrode contact.

Simulation time for each case was ~4 hours on a Dell PowerEdge R740xd server with 2x Intel^®^ Xenon^®^ Gold 6140 CPU with 18 cores and 1.5 TB RAM.

### D. VTA calculation

The VTA around each electrode contact was determined based on the concept of activation function (AF), calculated from eigenvalues of the Hessian matrix [6–8, 19]. We first interpolated the electric voltage on a regular 20 mm×20 mm×20 mm grid around the electrode with 0.1 mm resolution. The VTA was then determined by thresholding the AF along the orientation of maximal axonal excitability at each grid point. This optimal orientation can be determined through eigenvector decomposition of the Hessian matrix —the matrix of partial second spatial derivatives —of extracellular potential. Each eigenvalue of the Hessian matrix represents the second derivative of the electric potential along the respective eigenvector. The primary eigenvalue represents the largest second derivative, and thus the primary eigenvector is the orientation of maximal excitability.

We applied the activation threshold of 26.6 V/cm^2^ to determine the VTA based on dynamics of myelinated axons with 5.7 *μm* diameter and 70-ohm axoplasmic resistivity [19]. For each patient, we calculated the overlap of VTA with left and right NAcc.

## III. RESULTS

Table II gives the results of calculated VTA around left and right electrode contacts for each patient using the primary eigenvalue of Hessian matrix thresholded at 26.6V/cm^2^. We also calculated the overlap of the VTA with the NAcc for each patient and report pre-and postoperative Y-BOCS scores. The VTA was ~90 mm^3^ for patient 2 and ~66 mm^3^ for patient 1. The overlap of the VTA and the right NAcc was ~7.5 mm^3^ in patient 1 and 1.7 mm^3^ in patient 2, which is 2% and 0.3% of the whole volume of the right NAcc covered by the VTA. For the left NAcc, the overlap was modest and comparable in both patients (~3 mm^3^), which is 0.7% and 0.5% of the NAcc covered by the VTA for patient 1 and patient 2, respectively. The post-op Y-BOCS score showed a 68% improvement in patient 1 and a 40% improvement in patient 2, a trend that reflected the observed overlap between patients’ VTA and NAcc.

## IV. CONCLUSION

We report preliminary results showing association of patient outcome in response to DBS (here, % reduction in postoperative Y-BOCS) in two patients diagnosed with OCD who were treated with DBS of ventral capsule/ventral striatum. Among DBS targets for treating OCD, NAcc is associated with higher clinical improvement [5]. We used a patient-specific modeling approach to predict the VTA around DBS electrode contacts and found that the better clinical outcome was associated with a higher overlap of patient’s VTA and NAcc. Our results suggest that patient specific VTA prediction can be used to optimize stimulation profile to maximize the therapeutic effect and, in case of directional stimulation, minimize side effects.

## Figures and Tables

**Figure 2.**
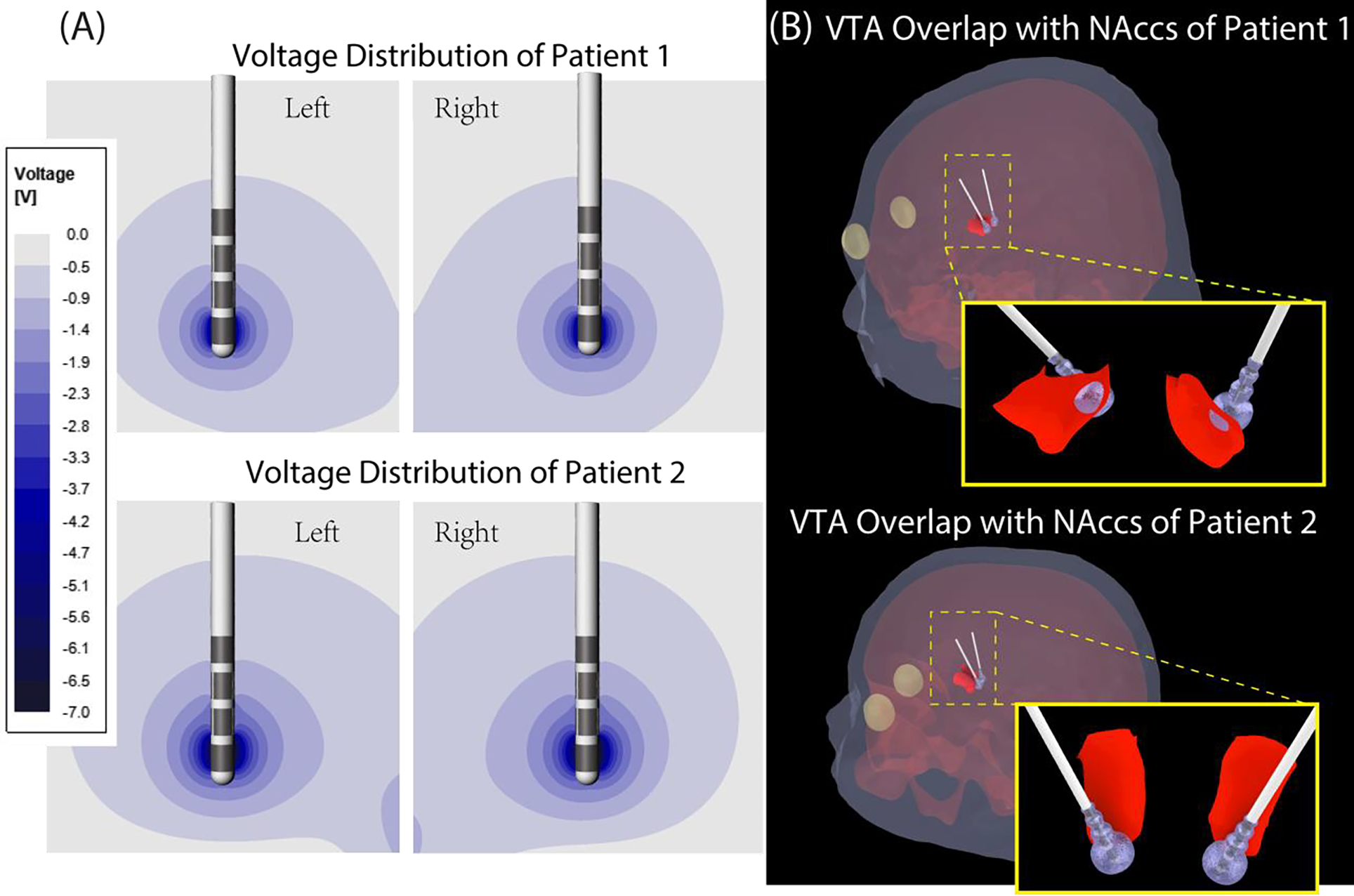
(A) Distribution of electric potential around implanted leads. (B) Shape and position of VTA (transparent lavender) with respect to NAcc(red).

**Table I:** Electrical properties of tissues used in each patient’s model

Dielectric parmeters of tissues in heterogeneous body			
Patient 1		Patient 2	
Tissue name	Conductivity [S/m] at 100 Hz	Tissue name	Conductivity [S/m] at 100 Hz
Average Body	0.400	Average Body	0.400
Skull	0.300	Skull	0.300
BrainGreyMatter	0.120	BrainGreyMatter	0.090
BrainWhiteMatter	0.058	BrainWhiteMatter	0.058
CerebroSpinalFluid	1.800	CerebroSpinalFluid	1.800
Ventricle	0.300	Ventricle	0.300

**Table II:** VTA [mm3] around Abbott lead 6172 ANS, calculated by thresholding the primary eigenvalue of Hessian matrix at 26.6V/cm2, and VTA [mm3] overlap with nacc. Pre- and post-op Y-BOCS scores are also given.

	VTA [mm^3^]: Field metric: Primary eigenvalue of Hessian matrix		VTA overlap with NAcc [mm^3^]		Pre-op YBOC	Post-op YBOC	% Improvement
	Left	Right	Left	Right			
Patient 1	66	66	3	7.5	31	10	68%
Patient 2	93	90	2	1.7	40	24	40%

## Clinical Relevance—

Patient-specific modeling of the volume of activated tissue can predict clinical outcomes and thus, can help to optimize DBS device programing to maximize therapeutic effects.

## References

[1] Ruscio AM, Stein DJ, Chiu WT, and Kessler RC, “The epidemiology of obsessive-compulsive disorder in the National Comorbidity Survey Replication,” Molecular psychiatry, vol. 15, no. 1, pp. 53–63, 2010. 18725912 10.1038/mp.2008.94 PMC2797569

[2] Anderson D and Ahmed A, “Treatment of patients with intractable obsessive—compulsive disorder with anterior capsular stimulation: Case report,” Journal of neurosurgery, vol. 98, no. 5, pp. 1104–1108, 2003. 12744372 10.3171/jns.2003.98.5.1104

[3] Sturm V , “The nucleus accumbens: a target for deep brain stimulation in obsessive–compulsive-and anxiety-disorders,” Journal of chemical neuroanatomy, vol. 26, no. 4, pp. 293–299, 2003. 14729131 10.1016/j.jchemneu.2003.09.003

[4] Coenen VA , “The medial forebrain bundle as a target for deep brain stimulation for obsessive-compulsive disorder,” (in English), Cns Spectrums, vol. 22, no. 3, pp. 282–289, Jun 2017, doi: 10.1017/S1092852916000286. 27268576

[5] Li N , “A unified connectomic target for deep brain stimulation in obsessive-compulsive disorder,” Nature communications, vol. 11, no. 1, pp. 1–12, 2020. 10.1038/s41467-020-16734-3 PMC7335093 32620886

[6] Anderson DN, Duffley G, Vorwerk J, Dorval AD, and Butson CR, “Anodic stimulation misunderstood: preferential activation of fiber orientations with anodic waveforms in deep brain stimulation,” Journal of neural engineering, vol. 16, no. 1, p. 016026, 2019. 30275348 10.1088/1741-2552/aae590 PMC6889961

[7] Anderson DN, Osting B, Vorwerk J, Dorval AD, and Butson CR, “Optimized programming algorithm for cylindrical and directional deep brain stimulation electrodes,” Journal of neural engineering, vol. 15, no. 2, p. 026005, 2018. 29235446 10.1088/1741-2552/aaa14b

[8] Duffley G, Anderson DN, Vorwerk J, Dorval AD, and Butson CR, “Evaluation of methodologies for computing the deep brain stimulation volume of tissue activated,” Journal of neural engineering, vol. 16, no. 6, p. 066024, 2019. 31426036 10.1088/1741-2552/ab3c95 PMC7187771

[9] Golestanirad L, Elahi B, Molina Arribere A, Mosig JR, Pollo C, and Graham SJ, “Analysis of fractal electrodes for efficient neural stimulation,” Frontiers in neuroengineering, vol. 6, p. 3, 2013. 23874290 10.3389/fneng.2013.00003 PMC3709379

[10] Golestanirad L, Izquierdo AP, Graham SJ, Mosig JR, and Pollo C, “Effect of realistic modeling of deep brain stimulation on the prediction of volume of activated tissue,” Progress In Electromagnetics Research, vol. 126, pp. 1–16, 2012.

[11] Jiang F, Nguyen BT, Elahi B, Pilitsis J, and Golestanirad L, “Effect of Biophysical Model Complexity on Predictions of Volume of Tissue Activated (VTA) during Deep Brain Stimulation,” in 2020 42nd Annual International Conference of the IEEE Engineering in Medicine & Biology Society (EMBC), 2020: IEEE, pp. 3629–3633. 10.1109/EMBC44109.2020.9175300 PMC10883758 33018788

[12] Bonmassar G, Golestanirad L, and Deng J, “Enhancing coil design for micromagnetic brain stimulation,” MRS advances, vol. 3, no. 29, pp. 1635–1640, 2018. 31105970 10.1557/adv.2018.155 PMC6519713

[13] Golestanirad L , “Solenoidal micromagnetic stimulation enables activation of axons with specific orientation,” Frontiers in physiology, vol. 9, p. 724, 2018. 30140230 10.3389/fphys.2018.00724 PMC6094965

[14] Nielsen JD , “Automatic skull segmentation from MR images for realistic volume conductor models of the head: Assessment of the state-of-the-art,” Neuroimage, vol. 174, pp. 587–598, 2018. 29518567 10.1016/j.neuroimage.2018.03.001

[15] Dale AM, Fischl B, and Sereno MI, “Cortical surface-based analysis. I. Segmentation and surface reconstruction,” Neuroimage, vol. 9, no. 2, pp. 179–94, Feb 1999, doi: 10.1006/nimg.1998.0395. 9931268

[16] Woolrich MW , “Bayesian analysis of neuroimaging data in FSL,” Neuroimage, vol. 45, no. 1 Suppl, pp. S173–86, Mar 2009, doi: 10.1016/j.neuroimage.2008.10.055. 19059349

[17] Fedorov A , “3D Slicer as an image computing platform for the Quantitative Imaging Network,” (in English), Magn Reson Imaging, vol. 30, no. 9, pp. 1323–1341, Nov 2012, doi: 10.1016/j.mri.2012.05.001. 22770690 PMC3466397

[18] Johnson H, Harris G, and Williams K, “BRAINSFit: mutual information rigid registrations of whole-brain 3D images, using the insight toolkit,” Insight J, vol. 57, no. 1, p. 2, 2007.

[19] Buhlmann J, Hofmann L, Tass PA, and Hauptmann C, “Modeling of a segmented electrode for desynchronizing deep brain stimulation,” Frontiers in neuroengineering, vol. 4, p. 15, 2011. 22163220 10.3389/fneng.2011.00015 PMC3233722

